# Antimicrobial susceptibility profile of uropathogens in Maluti Adventist Hospital patients, 2011

**DOI:** 10.4102/phcfm.v7i1.800

**Published:** 2015-05-12

**Authors:** Phillip Mubanga, Wilhelm J. Steinberg, Francois C. Van Rooyen

**Affiliations:** 1 Faculty of Health Sciences, Department of Family Medicine, University of the Free State, South Africa; 2 Faculty of Health Department Sciences, Department of Biostatistics, University of the Free State, South Africa

## Abstract

**Background:**

Urinary tract infections (UTIs) are amongst the most common infections encountered globally and are usually treated empirically based on bacterial resistance to antibiotics for a given region. Unfortunately in Lesotho, no published studies are available to guide doctors in the treatment of UTIs. Treatment protocols for Western countries have been adopted, which may not be applicable for this region.

**Aim:**

To determine the antimicrobial susceptibility profile of uropathogens in outpatients at the Maluti Adventist Hospital.

**Setting:**

The study was conducted at the outpatient department of the Maluti Adventist Hospital in Mapoteng, Lesotho.

**Methods:**

This was a prospective cross-sectional study using consecutive sampling of patients with clinical symptoms of UTI. Midstream urine samples were screened through chemistry and microscopy, then positive urine samples were cultured. The isolated uropathogens underwent antimicrobial susceptibility testing and inclusion continued until 200 culture samples were obtained. Descriptive statistics were used in the data analysis.

**Results:**

The top five cultured uropathogens were *Escherichia coli* (61.5%), *Staphylococcus aureus* (14%), *Pseudomonas* species (6.5%), *Enterococcus faecalis* (5.5%) and *Streptococcus agalactiae* (5%). The isolated uropathogens showed low sensitivity to cotrimoxazole (32.5% – 75.0%) and amoxicillin (33.2% – 87.5%) and high sensitivity to ciprofloxacin (84.0% – 95.1%) and nitrofurantoin (76.9% – 100%)

**Conclusion:**

In the Maluti setting, cotrimoxazole and amoxicillin should be avoided as first-line drugs for the empirical treatment of community-acquired UTI. We recommend the use of nitrofurantoin as first choice.

## Abstract (fr)

**Profile de risque antimicrobien d’uropathogènes chez les patients du Maluti Adventist Hospital, 2011.**

**Contexte:**

Les infections urinaires (UTI) sont les infections les plus courantes dans le monde et sont traitées en général de façon empirique en se basant sur la résistance bactériale aux antibiotiques dans une certaines région. Malheureusement il n’y a pas au Lesotho d’études publiées pour aider les médecins à traiter les UTI. On a adopté les programmes de traitement des pays occidentaux, ce qui n’est pas forcément applicable à cette région.

**Objectif:**

Déterminer le profile de risque antimicrobien d’uropathogènes chez les patients externes du Maluti Adventist Hospital.

**Lieu:**

L’étude a été faite au service de consultation externe du Maluti Adventist Hospital à Mapoteng, Lesotho.

**Méthodes:**

C’était une étude prospective transversale au moyen d’échantillons consécutifs des patients présentant des symptômes cliniques d’UTI. Les échantillons d’urine prélevés à mi-miction ont été testés chimiquement et au microscope, puis les échantillons d’urine positifs ont été cultivés. Les uropathogènes isolés ont été testés pour risques antimicrobiens et l’inclusion a été poursuivie jusqu’à l’obtention de 200 échantillons de cultures. On a utilisé les statistiques descriptives dans l’analyse des données.

**Résultats:**

Les cinq uropathogènes principaux cultivés étaient les espèces *Escherichia coli* (61.5%), *Staphylococcus aureus* (14%), *Pseudomonas* (6.5%), *Enterococcus faecalis* (5.5%) et *Streptococcus agalactiae* (5%). Les uropathogènes isolés ont montré une faible sensibilité au cotrimoxazole (32.5% – 75.0%) et à l’amoxicilline (33.2% – 87.5%) et une forte sensibilité à la ciprofloxacine (84.0% – 95.1%) et nitrofurantoine (76.9% – 100%)

**Conclusion:**

Dans le contexte du Maluti, il faut éviter le cotrimoxazole et l’amoxicilline comme médicaments de première intention pour le traitement empirique de l’UTI communautaire. Nous recommandons l’utilisation de la nitrofurantoine en premier choix.

## Introduction

Urinary tract infections (UTIs) are a major cause of morbidity worldwide. In a study done in Turkey on outpatient infections, UTIs were the second most common diagnosis after upper respiratory tract infections.^1^ A UTI occurs when there is the presence of pathogenic microorganisms along the urinary tract, involving one or more of the following: urethra, prostate, bladder and/or kidneys.^2^ Bacteria are by far the most common causative microorganisms of UTIs.

*Escherichia coli* is the leading bacterial uropathogen in the world.^3^ In a study on community-acquired infections, *E. coli* accounted for 68% of all the positive cultures for UTIs.^4^ This was followed by *Proteus mirabilis* (12%), *Staphylococcus aureus* (10%), *Enterococcus faecalis* (6%) and *Klebsiella aerogenes* (4%).^4^ These percentages and order of uropathogens after *E. coli* will vary from region to region, between men and women and between children and adults. For instance, in a study done at a tertiary hospital in South Africa, *E. coli* was also found to be the most cultured microorganism but it represented only 39% of all the uropathogens isolated. *Klebsiella* spp. followed at 20.8% and then *Enterococcus faecalis* at 8.2%.^5^ These differences highlight the importance of regional and institutional audits of antimicrobial susceptibility profiles.

Uncomplicated community-acquired UTIs are usually managed by the empirical prescription of antibiotics based on the bacterial resistance profile. This is done in order to avoid the unnecessary cost of doing a urine culture for every patient who presents with UTI symptoms. Guidelines recommend the empirical use of antibiotics if the bacterial resistance is less than 20%.^6,7^

The Ministry of Health and Social Welfare in Lesotho published the *Standard Treatment Guidelines for Lesotho* for the management of medical conditions in the country in 2006.^8^ For UTIs, the guidelines stipulate the use of cotrimoxazole, 80 mg or 400 mg, twice a day for seven days, as the first-line therapy for uncomplicated adult UTIs.

The use of amoxicillin is the other alternative recommended in the guideline. In severe cases, ampicillin by intravenous route is recommended and if patients do not respond, then ofloxacin or ciprofloxacin may be used.

### Aim and objectives

This study sought to determine the antimicrobial susceptibility profile of uropathogens responsible for community-acquired UTI in patients at the Maluti Adventist Hospital outpatient department, Lesotho. This information may be used to formulate a protocol for the appropriate and cost-effective empirical antibiotic treatment of uncomplicated UTIs in this resource-constrained setting.

The objectives of this study included:

- To establish the current profile of uropathogens responsible for community-acquired UTIs in patients at the Maluti Adventist Hospital outpatient department, Lesotho.
- To determine the antimicrobial susceptibility of these organisms.
- To formulate a suitable protocol for empirical antibiotic treatment of uncomplicated UTIs in this setting that is also cost-effective.

### Significance of the study

In the light of worldwide growing resistance of uropathogens to antibiotics, the variability in bacterial sensitivity profiles from region to region and the cost of re-treatment with its associated morbidity, it becomes imperative to establish a baseline and do periodic audits of the susceptibility profile of these pathogens so that treatment can be appropriate. Unfortunately for Lesotho, there are no published studies available to guide doctors in the treatment of UTIs. Empirical treatment protocols of other regions have been adopted, which may not be applicable.

## Research methods and design

### Study design

This was a prospective cross-sectional study on adult patients presenting with community-acquired uncomplicated UTIs, to the outpatient department of Maluti Adventist Hospital in Lesotho. A prospective study was chosen in order to eliminate the selection bias of a retrospective design done on past laboratory results only.

### Setting

The study was conducted in 2011 at the outpatient department of the Maluti Adventist Hospital in Mapoteng, Lesotho. This hospital is situated in the rural Berea District of Lesotho, about 70 km north of Maseru, the capital of Lesotho. Between July 2007 to June 2008, 29 125 outpatient visits were recorded. In the same period, 1298 patients were diagnosed with UTI based on clinical history and urine chemistry or microscopy. At Maluti Adventist Hospital's outpatient department, most UTIs are treated empirically with cotrimoxazole, amoxicillin or ciprofloxacin.

### Sample population and sampling strategy

Patients attending the outpatient department of Maluti Adventist Hospital with symptoms of UTI were eligible for recruitment. These included men and women older than 18 years who gave written consent for themselves and their urine samples to be used for the study.

Patients who had been hospitalised or had taken a course of antibiotics within the month prior to the study were excluded. Patients too ill to give consent, those with indwelling catheters and patients younger than 18 years of age were also excluded.

### Sample size

A sample size of 200 urine samples that were culture positives for UTI-causing microorganisms was thought to be sufficient to meet the objectives for this study. Each month, approximately 100 to 110 patients are seen at Maluti Adventist Hospital who have a positive urine microscopy or urinalysis suggestive of UTI. It was expected that more than 50% of these would give significant culture isolates. It was estimated that within a four- to six-month period, the needed 200 cultures would be obtained.

### Sample selection

Consecutive sampling was used. Patients qualifying for the study were selected for participation based on a clinical assessment for UTI. The symptoms included one or more of the following: dysuria, frequency, urgency and suprapubic pain. Potential candidates were informed about the study and asked to sign voluntarily consent. Thereafter, a thorough medical history and examination was done. They were then asked to give a midstream urine sample after a briefing on the technique.

This sequential recruitment of patients was done until the required number of culture-positive urine samples was obtained. Only one urine sample was obtained per patient.

### Intervention

Routine treatment procedures were initially followed at the outpatient department. But those participants whose culture samples showed that the antibiotics they had received where not effective, were changed to a susceptible antibiotic regime on follow-up.

### Data collection

Demographic data, including age, gender and UTI symptoms, results of microscopy and chemistry analyses and susceptibility testing, were captured on a data sheet.

Midstream urine samples from patients with UTI symptoms were collected in sterile containers and sent to the laboratory on the grounds of Maluti Adventist Hospital. Urine microscopy and chemistry were initially done to identify the urine samples for culture. Urine samples that qualified presented with one or more of the following: pyuria (> 4 white blood cells per ml) by microscopy, a positive leukocyte esterase result by chemistry and/or a positive nitrite result by chemistry.

The qualifying urine samples were inoculated on blood agar and MacConkey agar plates using a 0.001 mL inoculation loop. The inoculants were incubated aerobically at 37 °C for 24 hours. The presence of colony-forming units (CFUs), after incubation, greater than 10^5^ per mL was considered as significant bacteriuria, as per Kass criteria.^9^

For growths between 10^3^ to 10^5^ CFUs per mL, in which the source of the urine sample was a young woman with symptoms of cystitis or pyuria, the culture was also accepted as significant bacteriuria based on the Stamm criteria.^10^ The selected isolates were processed further for identification and antimicrobial susceptibility testing.

Identification was done on the basis of gram stains, morphology and biochemical features. The testing of the isolates for antimicrobial susceptibility was performed by means of the disc diffusion technique, according to the Clinical and Laboratory Standards Institute guidelines.^11^

The following four antibiotics were selected based on availability and cost implications: cotrimoxazole, amoxicillin, nitrofurantoin, and ciprofloxacin.

### Methodological and measurement errors

Potential methodological and measurement errors that could negatively affect data collection, analysis and interpretation of results were identified and remedial measures implemented:

- Improper urine collection technique leading to microbial contaminants and a subsequent false picture of the microbial profile was prevented by clearly instructing the patients on how to collect midstream urine.
- Inter-observer errors were minimised by using one microbiologist to perform the urinalysis, culture and antibiogram interpretation of the samples.

### Data analysis

Data collected during the study were analysed by the Department of Biostatistics at the Faculty of Health Sciences, University of the Free State. Descriptive statistics, namely means and standard deviations or medians and percentages, were used to calculate continuous data. Frequencies and percentages were used to calculate categorical data. The sensitivity of each uropathogen to the named antibiotics was determined in percentages. Charts and tables were used for clarity.

### Ethical considerations

Patients were asked to sign voluntary consent forms which were available in both English and Sesotho (the main language of Lesotho). Patients with positive urine culture results were informed and appropriate antibiotics prescribed by the treating doctor, if the empirical antibiotics had been inadequate.

Assurance was given to all patients that strict confidentiality would be maintained and that it would not prejudice their treatment if consent was denied.

Permission was obtained from the hospital management before going ahead with the study. The protocol was approved by the Ethics Committee of the Faculty of Health Science, University of the Free State (ETOVS number 21/07). Each patient was assigned a study number and this was recorded on the datasheet.

## Results

Urine samples collected from 609 patients with symptoms of UTI were sent to the laboratory for urine microscopy and chemistry testing. Of the 452 samples that qualified for culture, 200 samples yielded cultures and were further subjected to antibiotic sensitivity tests. The remaining 252 samples did not grow sufficient colonies or grew contaminants.

The patients of these 200 samples were between the ages of 18 to 84 years. Almost half (49.5%) were in the 20–29 year age group. The majority of patients were women (87.0%), of whom 28.3% were pregnant.

The pick-up ratio for UTI by symptom was as follows: dysuria 86.8%, suprapubic pain 63.1%, frequency 53.0%, urgency 41.9% and other symptoms 6.2%. The pick-ratio for UTI from urinalysis was urine microscopy 94.9%, urine leucocyte esterase 74.4% and urine nitrite 19.6%.

## Isolated uropathogens from cultured urine samples

More than two-thirds (61.5%) of the uropathogens isolated from the 200 cultured urine samples were identified as *E. coli* (Table 1).

**TABLE 1:** Uropathogens isolated from the cultured samples collected from patients with community-acquired urinary tract infections at the Maluti Adventist Hospital, Lesotho.

Uropathogens isolated	Variable	
	*n*	(%)
Total number of urine samples	609	100.0
Total number of urine samples with uropathogens	200	32.8
Uropathogens isolated (*n* = 200)
*Escherichia coli*	123	61.5
*Staphylococcus aureus*	28	14.0
*Pseudomonas* spp.	13	6.5
*Enterococcus faecalis*	11	5.5
*Streptococcus agalactiae*	9	4.5
*Proteus mirabilis*	8	4.0
*Staphylococcus epidermidis*	5	2.5
*Klebsiella aerogenes*	3	1.5
Other	10	5.0

The uropathogens showed high resistance (low sensitivity) to cotrimoxazole and amoxicillin. A much better susceptibility profile was seen for ciprofloxacin, but the uropathogens were most sensitive to nitrofurantoin (Table 2).

**TABLE 2:** Susceptibility profiles of the top five uropathogens isolated from patients with community-acquired urinary tract infections at the Maluti Adventist Hospital, Lesotho.

Top five uropathogens	% isolated from urine samples	Cotrimoxazole		Amoxicillin		Ciprofloxacin		Nitrofurantoin	
		*n*	% sensitivity	*n*	% sensitivity	*n*	% sensitivity	*n*	% sensitivity
*Escherichia coli*	61.5	40	32.5	41	33.3	117	95.1	117	95.1
*Staphylococcus aureus*	14.0	10	40.0	20	80.0	21	84.0	20	80.0
*Pseudomonas* spp.	6.5	4	30.8	6	46.2	11	84.6	10	76.9
*Enterococcus faecalis*	5.5	5	45.5	7	63.6	7	63.6	11	100.0
*Streptococcus agalactiae*	4.5	6	75.0	7	87.5	7	87.5	7	87.5

The sensitivity profile of this sample showed poor susceptibility to the commonly-used antibiotics, with acceptable susceptibility of the cultured microorganisms to ciprofloxacin and nitrofurantoin. The highest sensitivity was found for nitrofurantoin.

## Discussion

Community-acquired UTIs are usually treated empirically with antibiotics.^12^ Given this background, many doctors will send urine samples to the laboratory only after initial empirical treatment failure or for a complicated UTI, which may produce a bias in the laboratory records for urine culture and sensitivity. To avoid this bias, a prospective study, instead of a retrospective study, was done on past laboratory results.

*E. coli* was the most common uropathogen (61.5%) isolated from the cultured urine samples of this study population. This is similar to findings from studies done in other developing countries such as India (59%, 68%)^13,14^ and Madagascar (67%).^15^ A study done in South Africa reported the presence of *E. coli* in 75% of uncomplicated and 59% of complicated UTIs.^16^

*E. coli* had very low sensitivity levels to cotrimoxazole and amoxicillin. This may be a result of their widespread use in Lesotho. For example, cotrimoxazole is used for long-term infection prophylaxis in people with HIV.^8^

On the other hand, *E. coli* and the other uropathogens of this region had a high sensitivity to nitrofurantoin and ciprofloxacin. These antibiotics are not used as commonly in Lesotho compared with cotrimoxazole and amoxicillin. According to the *Standard Treatment Guidelines for Lesotho*,^8^ nitrofurantoin is not amongst the drugs listed for the treatment of community-acquired UTI and may be one of the reasons it is not commonly prescribed. *E. coli* sensitivity to ciprofloxacin and nitrofurantoin is less than 10% and therefore falls within the recommended guidelines for use in the empirical treatment of community-acquired UTI.^6,7^ However, there are concerns with the routine use of ciprofloxacin and other quinolones as first-line drugs, as resistance to these drugs may develop easily.^17,18^ Nitrofurantoin is also much cheaper than ciprofloxacin in terms of cost of treatment.^19^

It needs to be considered whether the antimicrobial susceptibility profile of uropathogens identified in this study can be used as representative of the general situation in Lesotho. Many patients at the Maluti Adventist Hospital outpatient department originate from outside the hospital catchment area, which can reach to Maseru and beyond.

### Limitations

Financial constraints limited the number of antibiotics used in testing the isolates for antimicrobial susceptibility.

In addition, the microbiologist was not available at all times and other laboratory staff had to stand in. This may have influenced inter-observer errors.

### Recommendations

For the Maluti Adventist Hospital catchment area, we recommend that cotrimoxazole and amoxicillin no longer be used as first-line drugs in the empirical treatment of adult community-acquired uncomplicated UTI. Nitrofurantoin should be the first choice of treatment, if there are no contraindications. It is also advised that the above recommendation be adopted for Lesotho in general until further evidence-based studies indicate otherwise. More studies need to be done in different parts of Lesotho to determine the overall antimicrobial susceptibility profile of uropathogens in the country and to keep tract of the uropathogen profile of this region.

## Conclusion

Bacteria causing community-acquired UTIs seen in patients at the Maluti Adventist Hospital outpatient department, show high resistance to cotrimoxazole and amoxicillin. This makes these antibiotics unsuitable as first-line treatment drugs. Bacterial resistance to nitrofurantoin and ciprofloxacin is very low, making them the preferable drugs. Yet costs make nitrofurantoin more favourable as a first-line drug in the empiric treatment of community-acquired UTI in adults.
